# Editorial: Circulating molecular biomarkers: next-generation tools for monitoring minimal residual disease in cancer patients

**DOI:** 10.3389/fonc.2023.1226974

**Published:** 2023-07-24

**Authors:** Zohreh Amoozgar, Mehdi Jaymand, Rana Jahanban-Esfahlan

**Affiliations:** ^1^ Edwin L. Steele Laboratories, Department of Radiation Oncology, Massachusetts General Hospital and Harvard Medical School, Boston, MA, United States; ^2^ Nano Drug Delivery Research Center, Health Technology Institute, Kermanshah University of Medical Sciences, Kermanshah, Iran; ^3^ Department of Medical Biotechnology, Faculty of Advanced Medical Sciences, Tabriz University of Medical Sciences, Tabriz, Iran

**Keywords:** tumor biomarkers, circulating tumor cells (CTCs), exosomes, cell-free DNA, liquid biopsy, cancer metastasis

Traditional cancer diagnosis relies on tissue biopsy, blood testing, and medical imaging, in which by the detection time, tumor size has reached several millimeters in size, and metastatic spread may have already begun. Plus, there is no accurate and timely way to monitor treatment response following surgery or drug treatment. Taking these shortcomings into account, new reliable tumor-specific biomarkers/tools that allow non-invasive early cancer detection, patient stratification for therapy, and monitoring of anti-cancer therapeutic regimens are highly demanded. The discovery of circulatory materials, including circulating tumor cells (CTCs), exosomes, cell-free DNA, and apoptotic bodies, and advances made in the fabrication of ultrasensitive biosensors that can capture and interrogate these rare yet valuable materials in different bodily fluids, plus advances in sequencing (NGS) methods and systems biology approaches have paved the path for the emergence of liquid biopsy and introduction of next-generation prognostic and diagnostic tools more powerful than traditional methods for better prediction and stratification and decision making for cancer patients at any stage from early detection to minimal residual disease monitoring (MRD) (1, 2).

In this Research Topic, several papers have reviewed the current status of circulating materials in cancer. Such that, Yi et al. shed light on the clinical use of exosomes as novel biomarkers for cancer diagnosis by focusing on exosome biogenesis, exogenous factors involved in exosome releasing from tumor cells, especially autophagy, hypoxia and pharmacology, and exosomal molecular cargoes as potential biomarkers in liquid biopsy for cancer diagnosis. From a different point of view, Nonaka reviewed the pontifical of chemically and genetically engineered exosomes for targeted therapy purposes and as next-generation therapeutics to overcome drug resistance. As current MRD assays are based on targeting somatic mutations, Johnston et al. emphasized the current state of both genetic and epigenetic liquid biopsies for MRD detection. Compared to genetic alterations, epigenetic changes are more frequent and universal in cancer, and cfDNA is enriched with epigenetic modifications, including DNA methylation alterations and fragmentation patterns. Finally, the authors discuss the emerging paradigm of genetic and epigenetic assays for monitoring treatment response, detecting the recurrence of disease, and informing adjuvant therapy.

Circulating proteins represent a major resource of MRD and disease monitoring. However, the scope of peptide-based circulating biomarkers is limited as they only contain one-fourth of the entire proteome. To surmount this, Tanuwidjaya et al. highlighted the importance of the Soluble Human Leukocyte Antigen (HLA) that presents peptides from the entire proteome on the cell surface. The authors reviewed the currently available tools to detect and quantify sHLA circulating biomarkers and further discussed these biomarkers’ challenges and future perspectives in a clinical setting.

In the sense of targeted drug delivery platforms for MRD, Mahmudi et al. highlighted the role of specific nanoparticle design formats and deciphered the role of tumor microenvironment penetrating chitosan nanoparticles to eliminate cancer relapse and MRD. This review focuses on the many advantages of chitosan as a suitable drug delivery system when combined with other biomaterials, to form hybrid and multitasking chitosan-based systems for numerous applications, in particular post-surgery implants (immunovaccines) and biosensors of tumor-derived circulating materials, as well as multimodal systems, to eliminate bulk tumors as well as lingering tumor cells to treat MRD and recurrent cancer.

In a research article, Sampathi et al. reported NanoporeMinION sequencing of clonal B cell-specific rearrangement of the (IGH) locus in cfDNA in 5 pediatric B-ALL patient samples as a biomarker for acute lymphoblastic leukemia (ALL) and MRD and its spread into the central nervous system (CNS). As a simple, rapid, and inexpensive assay, Nanopore IGH detection could monitor cell-free DNA for clinical diagnoses of MRD and CNS disease, even when diagnostic cell-count thresholds for MRD were not physically met.

Mi et al. reported a case of post-surgery and chemoradiotherapy laryngeal carcinoma patient with a high ratio of aneuploid circulating tumor endothelial cells (CTECs) to CTCs> 5 with rapid *de novo* development into pancreatic carcinomas. SE-iFISH detected a substantial amount of 107 non-hematological aneuploid circulating rare cells, including 14 CTCs (CD31-/CD45-) and 93 CTECs (CD31+/CD45-) five months before plasma increasing of CA19-9 and ten months prior to imaging-assisted diagnosis of *de novo* pancreatic cancer. Thus co-detection of aneuploid CD31- CTCs and CD31+ CTECs and the ratio CTECs to CTCs may help in the real-time evaluation of therapeutic efficacy, longitudinal monitoring of MDR, MRD, and reliable predicting post-therapy occurrence or distant metastatic recurrence of malignancy.

As an additional biomarker, Zha et al. reported the significance of FCGR3A as a new biomarker with potential predictive value for prostate cancer (PCa) using bioinformatics analysis. PPI and KEGG, as two bioinformatics tools, revealed the target gene cluster of FCGR3A, HAVCR2, CCR7, and CD28 with a high expression of FCGR3A and HAVCR2 in PCa tissue microarray (P<0.01). These two genes also showed a significant difference in BCR-free survival of FCGR3A and HAVCR2 as revealed by Kaplan-Meier analysis. Finally, TCGA clinical data analysis found that the expression of FCGR3A had a striking correlation with PCa clinicopathological features and tumor stage.

Wang et al. investigated the role of folate receptor-positive CTC count, lymphocyte count (LC), and derived neutrophil-to-lymphocyte ratio (dNLR) for diagnosing lung cancer relapse. Results from the clinicopathological features, routine blood tests, and CTC counts of the 69 patients indicated that a higher FR+-CTC count predicted a worse disease-or progression-free survival (DFS/PFS) in patients with lung cancer. Likewise, a retrospective study by Zhang et al. investigated the application of postoperative ctDNA for monitoring tumor recurrence in patients with non-small cell lung cancer (NSCLC) who underwent radical surgery operation followed by adjuvant therapy. Defining a positive molecular residue disease (MRD) as having at least one true shared mutation in both plasma and tissue samples from the same patient indicated that a lower recurrence-free survival (RFS) was accompanied by positive postoperatively ctDNA and MRD presence was a strong predictor of disease recurrence. The ctDNA-based MRD was a strong prognostic of RFS higher than all other clinicopathological variables and even the traditional TNM staging.

Circulatory materials and liquid biopsy are newly emerged discoveries with multiple FDA-approved tests. As such, Clonoseq explores clonally expanded VDJ sequences in blood and bone marrow-derived gDNA samples to identify and detect as few as one leukemic blast per million cells in blood cancer patients, with a sensitivity of 6.77×10^−7^ (3, 4). Also, in 2020, FoundationOne’s Liquid CDx cfDNA was introduced to detect cfDNA with specific mutations in solid tumors (5). Also, in ALL, cfDNA assays can detect bone marrow relapse 30 days earlier than the standard flow cytometry-based methods. Altogether, these data pinpoint the critical role of circulating materials in the future of diagnostic tools for cancer monitoring and control of MRD. Liquid biopsy and novel biomarker discovery are expected to become the forefront of the next-coming diagnostic toolkits and pave the way for realizing personalized medicine. However, the high cost is the limiting factor for the broad applicability of these tests, given their high sensitivity, specificity, and diagnostic value compared to traditional sample biopsy coupled with NGS, biosensing devices, and systems biology approaches yet continue to reveal and offer new opportunities by introducing novel biomarkers to predict and win the game in the battle of cancer as well as other diseases.

## Author contributions

ZA, MJ, and RJ-E have equally written and edited the editorial. All authors contributed to the article and approved the submitted version.
